# Perioperative Management of Elderly patients (PriME): recommendations from an Italian intersociety consensus

**DOI:** 10.1007/s40520-020-01624-x

**Published:** 2020-07-10

**Authors:** Paola Aceto, Raffaele Antonelli Incalzi, Gabriella Bettelli, Michele Carron, Fernando Chiumiento, Antonio Corcione, Antonio Crucitti, Stefania Maggi, Marco Montorsi, Maria Caterina Pace, Flavia Petrini, Concezione Tommasino, Marco Trabucchi, Stefano Volpato, Società Italiana di Anestesia Analgesia Rianimazione e Terapia Intensiva (SIAARTI), Società Italiana di Gerontologia e Geriatria (SIGG), Società Italiana di Chirurgia (SIC), Società Italiana di Chirurgia Geriatrica (SICG) and Associazione Italiana di Psicogeriatria (AIP)

**Affiliations:** 1 Fondazione Policlinico Universitario A. Gemelli IRCCS, Rome, Italy; 2 Università Cattolica del Sacro Cuore, Rome, Italy; 3 Policlinico Universitario Campus Biomedico, Rome, Italy; 4 Past Director Geriatric Surgery Area and Anaesthesia Dpt., INRCA, Italian National Research Centre on Aging, Ancona, Italy; 5 Università degli Studi di Padova, Padua, Italy; 6 ASL Salerno, Salerno, Italy; 7 Monaldi-Ospedale Dei Colli, Naples, Italy; 8 CNR, Institute of Neuroscience, Aging Branch, Padua, Italy; 9 Humanitas University and Research Hospital IRCCS, Milan, Italy; 10 Università degli Studi “Luigi Vanvitelli”, Naples, Italy; 11 Università degli Studi G.d’Annunzio, Chieti, Italy; 12 Università degli Studi di Milano, Milan, Italy; 13 Università degli Studi di Tor Vergata, Rome, Italy; 14 Università degli Studi di Ferrara, Ferrara, Italy

**Keywords:** Older patients, Anesthesia, Surgery, Perioperative care, Frail older, Analgesia, Comprehensive geriatric assessment

## Abstract

**Background:**

Surgical outcomes in geriatric patients may be complicated by factors such as multiple comorbidities, low functional performance, frailty, reduced homeostatic capacity, and cognitive impairment. An integrated multidisciplinary approach to management is, therefore, essential in this population, but at present, the use of such an approach is uncommon. The Perioperative Management of Elderly patients (PriME) project has been established to address this issue.

**Aims:**

To develop evidence-based recommendations for the integrated care of geriatric surgical patients.

**Methods:**

A 14-member Expert Task Force of surgeons, anesthetists, and geriatricians was established to develop evidence-based recommendations for the pre-, intra-, and postoperative care of hospitalized older patients (≥ 65 years) undergoing elective surgery. A modified Delphi approach was used to achieve consensus, and the strength of recommendations and quality of evidence was rated using the U.S. Preventative Services Task Force criteria.

**Results:**

A total of 81 recommendations were proposed, covering preoperative evaluation and care (30 items), intraoperative management (19 items), and postoperative care and discharge (32 items).

**Conclusions:**

These recommendations should facilitate the multidisciplinary management of older surgical patients, integrating the expertise of the surgeon, the anesthetist, the geriatrician, and other specialists and health care professionals (where available) as needed. These roles may vary according to the phase and setting of care and the patient’s conditions.

## Introduction

People over 65 years of age currently account for 23% of the Italian population [1], and in 2017, approximately 45% of surgical interventions were performed in this age group. The traditional clinical approach, focusing on a single disease, is often insufficient in geriatric patients, for many reasons including multiple comorbidities, low functional performance, frailty, reduced homeostatic capacity, and cognitive impairment. Geriatric surgical patients, therefore, require integrated care from the preoperative evaluation throughout the perioperative period. However, although multidisciplinary care models for geriatric patients, such as the orthogeriatric model [2], are long established, this integrated approach appears to be rarely used in older patients undergoing other major surgeries. For this reason, the PriME (Perioperative Management of the Elderly) project has been developed by a multidisciplinary panel of anesthetists, surgeons, and geriatricians, aiming to highlight the specific needs of older surgical patients, and to propose recommendations for the integrated care of geriatric surgical patients.

## Materials and methods

PriME is a collaborative initiative of SIAARTI (Italian Society of Anesthesia, Analgesia, Intensive Care and Intensive Care), SIGG (Italian Society of Gerontology and Geriatrics), SIC (Italian Society of Surgery), Society of Geriatric Surgery (SICGe), and AIP (Italian Association of Psychogeriatrics). These societies appointed a 14-member Expert Task Force, which met in September 2018 to define the scope of the project, identify key issues, and agree consensus methods. It was decided that the focus should be on hospitalized patients aged > 65 years undergoing elective surgery; three main areas for investigation were identified (preoperative, intraoperative, and postoperative care), and corresponding subcommittees appointed. A modified Delphi approach was used to achieve consensus, and the U.S. Preventive Services Task Force system [3] used to rate the strength of recommendations (Table 1) and level of certainty/quality of evidence (Table 2).

**Table 1:** US Preventive Services Task Force grading of strength of recommendations (3)

Grade	Definition	Suggestion for practice
A	The USPSTF recommends the service. There is high certainty that the net benefit is substantial	Offer or provide this service
B	The USPSTF recommends the service. There is high certainty that the net benefit is moderate or there is moderate certainty that the net benefit is moderate to substantial	Offer or provide this service
C	The USPSTF recommends selectively offering or providing this service to individual patients based on professional judgment and patient preferences. These is at least moderate certainty that the net benefit is small	Offer or provide this service for selected patients depending on individual circumstances
D	The USPSTF recommends against the service. There is moderate or high certainty that the service has no net benefit or that the harms outweigh the benefits	Discourage the use of this service
I	The USPSTF concludes that the current evidence is insufficient to assess the balance of benefits and harms of the service. Evidence is lacking, of poor quality, or conflicting, and the balance of benefits and harms cannot be determined	Read the clinical considerations section of USPSTF Recommendation Statement. If the service is offered, patients should understand the uncertainty about the balance of benefits and harms

**Table 2:** Grading of quality of evidence ( modified from US Preventive Services Task Force (3)

Quality of evidence	Description
High (A)	The available evidence usually includes consistent results from a multitude of well-designed, well-conducted, studies in representative care populations. These studies assess the effects of the service on the desired health outcomes. Because of the precision of findings, this conclusion is, therefore, unlikely to be strongly affected by the results of future studies. These recommendations are often based on direct evidence from clinical trials of screening, treatment or behavioral interventions. High-quality trials designed as “pragmatic” or “effectiveness” trials are often of greater value in understanding external validity
Moderate (B)	The available evidence is sufficient to determine the effects of the service on targeted health outcomes, but confidence in the estimate is constrained by factors such as: The number, size, or quality of individual studies in the evidence pool Some heterogeneity of outcome findings or intervention models across the body of studies Mild-to-moderate limitations in the generalizability of findings to routine care practice. As more information becomes available, the magnitude or direction of the observed effect could change, and this change may be large enough to alter the conclusion
Low (C)	The available evidence is insufficient to assess effects on health outcomes. Evidence is insufficient because of: The very limited number or size of studies Inconsistency of direction or magnitude of findings across the body of evidence Critical gaps in the chain of evidence Findings are not generalizable to routine care practice A lack of information on prespecified health outcomes Lack of coherence across the linkages in the chain of evidence. More information may allow an estimation of effects on health outcomes

Based on a literature review, each subcommittee developed a list of topics, and proposed specific recommendations with supporting evidence for each topic. Key issues were discussed at a meeting in January 2019, after which a comprehensive document was circulated, and subjected to three rounds of revision. Statements for which consensus was achieved (defined as > 70% agreement with < 15% disagreement) were than resubmitted to the Expert Task Force at a Consensus Conference in July 2019, where recommendations and supporting evidence were reviewed and discussed by the entire group, to achieve a final consensus. Subsequently, a draft report was prepared and sent to the Experts for modification and comment. Each author approved the final version prior to submission.

A complete list of all recommendations is included in Table 3.

**Table 3:** Summary of recommendations

Statement	Quality of evidence	Strength of recommendation
**Preoperative assessment and optimization**		
We recommend that the Timed Up-and-Go (TUG) test be performed for all patients. In case of pathological values (> 20 s), Comprehensive Geriatric Assessment (CGA) is necessary	Moderate	A
We suggest CGA for all older patients	Moderate	B
We recommend that this preoperative assessment be the responsibility of the anesthetist, in collaboration with a geriatrician if available, who together share assessments and appropriate pathways with the surgical staff	Moderate	A
We recommend that functional reserves and independence be evaluated before the intervention	Moderate	A
We suggest using multiparametric frailty scales (e.g., Fried Score or Edmonton Frailty Score) to identify areas where preoperative optimization is necessary	Low	B
We recommend a systematic prehabilitation strategy to improve functional status and increase the organic functional reserve	Low	A
We recommend a cardiopulmonary exercise test before major surgery (e.g., cardiovascular or thoracic)	Low	A
We recommend that the risk of falls be assessed, especially in older patients with reduced mobility, postural hypotension, or risk of syncope, and that preventive measures be adopted	Low	A
Visual and auditory aids must always be readily available and accessible to the patient, and should be removed only when their use conflicts with other needs	Low	A
We recommend cognitive assessment (e.g., Clock test, AMT, and MMSE) of all patients aged > 65 years, even in the absence of a history of cognitive decline We recommend a second-level specialist neurocognitive assessment for patients with pathological test scores	Moderate	A
We recommend that the relative implications of comorbidities, and chronic or degenerative pathologies, for the response to surgery be recognized	Low	A
It is recommended that the cardiovascular risk assessment includes: • Functional capacity and, in case of major surgery, cardiac stress test (when indicated) • A cardiac risk index (e.g., Lee Index) or a surgical risk calculator that includes age and comorbidities (ACS-NSQIP Surgical Risk Calculator) • Risk of cardiac complications associated with the type of surgical intervention	Moderate	A
Before surgery, we recommend that patients at risk of venous thromboembolism be identified and appropriate perioperative prophylaxis be established	Moderate	A
We recommend that risk factors for respiratory complications be assessed and reduced where possible (e.g., abstention from smoking and optimization of therapies)	Moderate	A
We suggest the use of risk scores for postoperative pulmonary complications (ARISCAT)	Moderate	B
We recommend that a Patient Blood Management (PBM) strategy be implemented, including hemoglobin and iron optimization, predeposit autologous blood collection, and surgical and anesthetic strategies that reduce blood loss	Moderate	A
We recommend that hemoglobinemia be assessed in all geriatric patients, with particular attention to those aged > 80 years	Moderate	A
We recommend assessment of hemoglobinemia and iron metabolism in all geriatric patients who are candidates for surgery with expected blood loss > 500 ml, or who have fatigue, neoplastic disease, associated cardiovascular, respiratory or renal disease, resting tachycardia or pallor	Moderate	A
We recommend accurate estimation of renal function by calculating eGFR using the CKD-EPI equation	Moderate	A
We recommend evaluation of nutritional status and correction of any deficiency, especially before major surgery	Moderate	A
We recommend that albuminemia be assessed in all older surgical patients, especially those with hepatic comorbidity, multiple comorbidities, recent major pathology or suspected malnutrition, or candidates for major surgery	Moderate	A
In candidates for major surgery with organ failure, we recommend an estimation of hydration and volume status with an instrumental method (e.g., ultrasound estimation or bioimpedentiometry)	Moderate	A
We recommend pre- and postoperative nutritional support (enteral or parenteral), according to the level of undernutrition and/or feeding possibilities for patients at severe nutritional risk	Moderate	A
It is recommended that the pharmacological history must be extended to include all drugs used by the patient, including over-the-counter and herbal medicines	Low	A
If the patient is taking inappropriate medications (e.g., according to STOPP criteria), we recommend prudent withdrawal of these medications	Low	A
To reduce the incidence of postoperative delirium, we recommend: • Identifying predisposing and precipitating risk factors early • Adapting surgical and anesthetic techniques • Avoiding medications that promote postoperative delirium • Using opioid-free anesthesia or low-dose opioid anesthesia • Monitoring for delirium (CAM, 4AT)	Moderate	A
Every older patient should undergo a standardized pain history and physical examination For patients with cognitive disorders, we recommend the use of specific scales (PAINAD, NOPPAIN) for the evaluation of pain	Low	A
We suggest screening for depression using validated scales (e.g., the Geriatric Depression Scale), and treatment where possible	Low	B
Where possible, we suggest preventive counseling (goal setting, advanced directives) in selected cases	Low	B
It is recommended that the availability of family and social support be investigated during the preoperative assessment to allow planning of substitutive support measures	Low	A
**Intraoperative management**		
When positioning an older patient on the operating table, we suggest that attention be paid to conditions of the skin (e.g., atrophy, injury) and the musculoskeletal system (e.g., bone deformities, joint stiffness, and presence of prostheses)	Low	B
It is recommended that positioning be adjusted according to the patient's problems, taking care to place adequate padding at bony prominences	Low	A
It is recommended that the choice of anesthesia (technique/drugs/dosage) be individualized based on the characteristics of the patient and the type of intervention, in order to reduce the incidence of postoperative delirium and facilitate recovery	Moderate	A
We recommend dose adjustment to avoid overdose, adverse hemodynamic effects, or inadequate depth of narcosis	Moderate	A
For induction and maintenance of general anesthesia with propofol, we recommend that the dosage be reduced by 20–50% in older patients	Moderate	A
For halogenated anesthetics, we recommend that the minimal alveolar concentration be calculated according to patient age	Moderate	A
Because the effect of anesthetics on the central nervous system is age-dependent, we recommend that halogenated and intravenous anesthetic dosages be modulated using an anesthesia depth monitoring system	Moderate	A
During general anesthesia, we recommend EEG-based monitoring to avoid excessive anesthesia depth, which is associated with increased risk of postoperative delirium	High	A
It is recommended that EEG-based monitoring is extended to procedures performed under sedation	High	A
In patients undergoing general anesthesia with neuromuscular block, we recommend that neuromuscular function be monitored quantitatively, and its complete recovery (train-of-four ratio > 0.9) be facilitated at the end of the intervention	Moderate	A
We recommend that residual neuromuscular block always be antagonized	Moderate	A
We recommend the use of sugammadex when complete and fast recovery of rocuronium-induced neuromuscular block is required	Moderate	A
We recommend the use of sugammadex when anticholinesterases are ineffective for reversing rocuronium-induced neuromuscular block	Moderate	A
We recommend body-temperature monitoring and active warming of the patient, preferably with a forced-air system, during the pre-, intra- and postoperative periods	High	A
If forced-air heating is only partially efficacious (e.g., during prolonged open abdominal surgery), we suggest that warm intravenous fluids be administered	High	B
We recommend adequate monitoring to maintain a “near-zero” fluid balance	High	A
We recommend that transfusion in geriatric patients follows a restrictive transfusion strategy (red blood cell transfusion threshold: Hb < 8 g/dl)	High	A
We recommend red blood cell transfusion when symptoms of intraoperative hypoxia and/or lactic acidosis and hemorrhage are present, regardless of the severity of anemia	High	A
We suggest using minimally invasive techniques in older patients, to reduce the endocrine/metabolic response to stress and improve postoperative outcomes	Moderate	B
During laparoscopy, we recommend: • Avoiding exaggerated or prolonged Trendelenburg or anti-Trendelenburg positions • Avoiding unjustified prolongation of surgical times • Using the lowest possible intraperitoneal pressure (< 12 mmHg), to minimize the negative cardiovascular and respiratory effects caused by pneumoperitoneum • Administering deep neuromuscular blockade, to allow the use of low working pressures	Moderate	A
**Postoperative care**		
In the postoperative period, we recommend: • Optimal postoperative pain control • Early mobilization and walking • Early resumption of feeding • Conservation of the sleep–wake rhythm • Reducing use of nasogastric tube and bladder catheters • Antithrombotic prophylaxis	Moderate	A
We recommend the use of Enhanced Recovery Pathways for older patients, according to the type of surgery performed	Moderate	A
It is recommended that prevention, recognition, and treatment of postoperative delirium must be an objective of the multidisciplinary team	Moderate	A
We recommend that patients at risk for POD be monitored with validated diagnostic tools such as the CAM or 4AT, starting when they wake from anesthesia and continuing for 5 days thereafter	Moderate	A
Because of the high risk (e.g., of aspiration pneumonia), we recommend the use of adequate prophylaxis against postoperative nausea and vomiting, and semi-Fowler's decubitus position	Low	A
Personalized prevention and treatment of postoperative pain are mandatory. We recommend a multimodal approach or, when possible, locoregional or plane blocks (e.g., TAP block)	Low	A
We suggest encouraging use of the non-pharmacological components of the multimodal approach	Low	B
We recommend periodic evaluation of oxygen saturation and respiratory rate in the postoperative period	Moderate	A
We recommend that arterial blood gas analysis be used when conditions interfere with percutaneous oximetry (e.g., shivering, tremor, cold skin, hyperthermia, hypotension, advanced heart failure, high fever, atrial fibrillation, or other arrhythmias)	Moderate	A
We suggest that older patients should be treated with lung expansion techniques, such as deep breathing exercises, incentive spirometry, or, when indicated, with non-invasive ventilation	Moderate	B
To prevent postoperative cardiac complications, we recommend monitoring (continuously in selected cases) and maintenance of cardiovascular measures (e.g., heart rate, arterial pressure) within baseline values	High	A
We suggest the use of graduated compression stockings or intermittent pneumatic compression, when indicated	High	B
For prevention of renal damage, we recommend that adequate hydration be maintained, and hypovolemia and/or hypotension be avoided	High	A
We recommend caution in the use of potentially nephrotoxic drugs or drug combinations with a high risk of nephrotoxicity, and in the administration of contrast media for radiological procedures	High	A
We recommend that urinary catheters be used only when essential, and be removed as soon as possible	High	A
We recommend to adopt strategies to prevent urinary tract infections before, during and after insertion of a urinary catheter	High	A
We do not recommend complementary strategies (such as the use of alpha-blockers in men) to promote spontaneous urinary function after catheter removal	High	D
It is recommended that older patients undergo daily assessment of caloric intake and water balance	Moderate	A
We recommended that swallowing should be evaluated, and the presence of oral lesions excluded in patients with signs and symptoms of dysphagia or a history of aspiration pneumonia	Moderate	A
We suggest that all older patients are seated during meals and for an hour after eating	Moderate	B
It is recommended that nutritional supplementation be given in patients with malnutrition or inadequate nutrition	Moderate	A
It is recommended that dental prostheses, if used, are readily available and easily accessible	Moderate	A
Strategies to prevent and treat pressure injuries are recommended in patients at risk	Moderate	A
We recommend guideline-consistent antimicrobial prophylaxis in older patients, considering antibiotic pharmacodynamics and pharmacokinetics to avoid overdoses and drug-related adverse events	Moderate	A
We recommend careful and prolonged assessment of blood glucose in older patients with or without diabetes. Insulin is the treatment of choice for the management of hyperglycemias	Moderate	A
We suggest that blood glucose values up to 180 mg/dl are acceptable in critical postoperative patients	Moderate	B
During the surgical planning, we recommend evaluating the probability of the patient returning home at the end of the hospital stay	Moderate	A
For patients with functional deterioration and/or weak social networks, we recommend that the discharge plan, including outpatient visits and medications, should be organized in advance in collaboration with the geriatrician; ideally, a Geriatric Evaluation Unit should be available	Moderate	A
Before hospital discharge, we recommend re-evaluating and adjusting drug therapy according to home therapy, the surgery performed, comorbidities, and any new pathological changes	Moderate	A
We recommend that the patient and caregiver be provided with resources to deal with problems that may arise at home, and with patient management contacts and references (telephone/e-mail)	Moderate	A

## Preoperative assessment and optimization

### Comprehensive geriatric assessment (CGA)

**Table:** 

Statement	Quality of evidence	Strength of recommendation
We recommend that the Timed Up-and-Go (TUG) test be performed for all patients. In case of pathological values (> 20 s), Comprehensive Geriatric Assessment (CGA) is necessary	Moderate	A
We suggest CGA for all older patients	Moderate	B
We recommend that this preoperative assessment be the responsibility of the anesthetist, in collaboration with a geriatrician if available, who together share assessments and appropriate pathways with the surgical staff	Moderate	A
We recommend that functional reserves and independence be evaluated before the intervention	Moderate	A

The preoperative assessment should evaluate the patient's health status to assess the surgical risk, increase functional reserves, manage vulnerability, and anticipate, minimize, or prevent possible complications. This requires a team-based approach throughout the entire care pathway [4]. The anesthetist should guide the team in the perioperative phase, and the geriatrician should take the lead thereafter.

Comprehensive Geriatric Assessment (CGA) is a multimodal, multidisciplinary, process aimed at identifying care needs, planning care, and improving clinical and functional outcomes for older people [5]. This process includes both clinical data and functional measures of cognitive, psychological, nutritional, and behavioral status, and evaluation of social or family support. The aims are to improve diagnostic accuracy, optimize medical treatment, improve medical outcomes, optimize the home environment, minimize unnecessary service use, and arrange long-term management.

CGA and frailty evaluation are extremely useful in surgical risk evaluation in older patients, and in making decisions about surgery [4, 6–8]. However, evidence from randomized-controlled trials, large systematic reviews, and meta-analyses suggests that the effectiveness of CGA may vary according to the healthcare setting. For example, home-based and in-hospital CGA programs have consistently been shown to improve health outcomes, whereas evidence is less conclusive for post-hospital discharge CGA programs, outpatient CGA consultation, and CGA-based inpatient geriatric consultation services [9]. The effectiveness of CGA may be reduced in patients with specific clinical conditions, such as frailty, cancer, or cognitive impairment [9].

CGA is recommended in all geriatric surgical patients, and is mandatory in those with a timed up-and-go (TUG) test result > 20 s. However, because CGA is time-consuming and sometimes difficult to apply in clinical practice, involvement of hospital medical services to create specific management pathways is needed to implement this approach.

#### Frailty

**Table:** 

Statement	Quality of evidence	Strength of recommendation
We suggest using multiparametric frailty scales (e.g., Fried Score or Edmonton Frailty Score) to identify areas where preoperative optimization is necessary	Low	B

Frailty is an age‐related decline in physiological reserves that results in diminished resilience, loss of adaptive capacity, and increased vulnerability to stressors. Signs of frailty include unintentional weight loss, self-reported exhaustion, slow walking speed, weak grip strength, and low physical activity level [10]. Frailty is strongly predictive of adverse postoperative outcomes, including medical complications, prolonged hospitalization, institutionalization, readmission, and short‐ and long‐term mortality [11, 12]. Hence, the American College of Surgeons National Surgical Quality Improvement Program/American Geriatrics Society (ACS-NSQIP/AGS) 2012 Guidelines for the optimal pre‐operative assessment of geriatric surgical patients have identified frailty assessment as a critical component in the pre‐operative assessment [13], and this was reinforced by a 2015 consensus statement [14].

The Edmonton Frail Scale (EFS) [15] is a 17‐point scale incorporating 10 clinical and functional domains. It is not time-consuming, and can be easily used by non-geriatricians.

#### Prehabilitation strategy

**Table:** 

Statement	Quality of evidence	Strength of recommendation
We recommend a systematic prehabilitation strategy to improve functional status and increase the organic functional reserve	Low	A
We recommend a cardiopulmonary exercise test before major surgery (e.g., cardiovascular or thoracic)	Low	A
We recommend that the risk of falls be assessed, especially in older patients with reduced mobility, postural hypotension or risk of syncope, and that preventive measures be adopted	Low	A
Visual and auditory aids must always be readily available and accessible to the patient, and should be removed only when their use conflicts with other needs	Low	A

Patients with functional impairment are at increased risk of postoperative complications [16]. Appropriate measures, where needed, should, therefore, be taken to increase functional reserves.

##### Prehabilitation

Patients with functional deficits in activities of daily living, or difficulties with mobility, should be referred to an occupational or physical therapist. Such patients may benefit from preoperative physical conditioning (prehabilitation) to enhance their capacity to withstand surgical stress and promote postoperative recovery [17]. Multimodal prehabilitation, including home exercise, nutrition assessment, and pain management, improves postoperative functional outcomes in older surgical patients [18].

##### Cardiopulmonary exercise testing

Cardiopulmonary exercise testing objectively measures aerobic fitness or functional capacity. It provides an individualized estimate of patient risk that can be used to predict postoperative morbidity and mortality, inform decision-making, determine the most appropriate perioperative care environment, diagnose unexpected comorbidities, optimize medical comorbidities preoperatively, and direct individualized preoperative exercise programs [19].

##### Falls

Falls are the primary cause of unintentional injury, and a leading cause of death, in older adults. Limited mobility and falls lead to functional decline, hospitalization, institutionalization, and increased health care costs [20]. A history of falls within 6 months before surgery is associated with increased rates of postoperative complications, discharge to a rehabilitation facility, and hospital readmission [21]. Hence, it is recommended that the risk of falls be assessed preoperatively, and appropriate preventive measures taken, particularly in patients with reduced mobility, postural hypotension, or risk of syncope. The risk of falls can be assessed with the TUG test [22].

##### Sensory deficits and use of functional aids

Concomitant sensory and cognitive impairment is common in older individuals [23], and is an independent risk factor for postoperative death and complications [24]. Multimodal interventions including elements addressing visual or hearing impairment can significantly reduce the prevalence and duration of delirium in older hospitalized patients [25]. Hence, the NSQIP/AGS guidelines state that strategies for the prevention of postoperative delirium may include functional aids for visual and hearing impairment, and that such aids should be made available to the patient after surgery [13]. The patient’s level of comprehension should be systematically checked at critical steps of the care process.

##### Cognitive function

**Table:** 

Statement	Quality of evidence	Strength of recommendation
We recommend cognitive assessment (e.g., Clock test, AMT, and MMSE) of all patients aged ≥ 65 years, even in the absence of a history of cognitive decline We recommend a second-level specialist neurocognitive assessment for patients with pathological test scores	Moderate	A

In Europe, 35–50% of persons older than 65 years have mild cognitive impairment (MCI) or dementia [26, 27]. However, cognitive impairment—particularly MCI—is often undiagnosed [28‒30]. Postoperative delirium (POD) and postoperative cognitive dysfunction (POCD) affect 20–80% and 12–15%, respectively, of geriatric surgical patients; preexisting cognitive impairment is a risk factor for both conditions, as well as being a predictor or modifier of postoperative outcomes [31‒33]. Routine screening for cognitive impairment should, therefore, be included in the preoperative evaluation, even in patients with no history of cognitive decline. Basic cognitive tests, such as the Clock drawing test, the Abbreviated Mental Test, or the Mini-Mental State Examination (MMSE), can be used for screening; specialist investigation is required in patients with equivocal findings.

### Comorbidities

**Table:** 

Statement	Quality of evidence	Strength of recommendation
We recommend that the relative implications of comorbidities, and chronic or degenerative pathologies, for the response to surgery be recognized	Low	A

The combination of aging and comorbidities is the principal factor reducing tolerance to surgical stress in older patients [4]. Comorbidities increase markedly with age, largely due to increasing rates of chronic conditions [10]. The most common comorbidity in older patients is hypertension, which affects 45–50% of patients over 70 years of age, followed by coronary artery disease (CAD) (35%); other common comorbidities include diabetes (12–15%) and chronic obstructive pulmonary disease (COPD) (9%) [34]. Comorbidities are strongly associated with increased surgical and postoperative risks, and increased health care costs [35].

#### Cardiovascular

**Table:** 

Statement	Quality of evidence	Strength of recommendation
It is recommended that the cardiovascular risk assessment includes: • Functional capacity and, in case of major surgery, cardiac stress test (when indicated) • A cardiac risk index (e.g., Lee Index) or a surgical risk calculator that includes age and comorbidities (ACS-NSQIP Surgical Risk Calculator) • Risk of cardiac complications associated with the type of surgical intervention	Moderate	A
Before surgery, we recommend that patients at risk of venous thromboembolism be identified and appropriate perioperative prophylaxis established	Moderate	A

Age-related changes in the cardiovascular and autonomic nervous systems reduce cardiac responsiveness to stress [36]. Guidelines for the evaluation of cardiac risk published by the American College of Cardiology (ACC) and the American Heart Association (AHA) [37] recommend preoperative cardiac testing only if the results will change clinical management, and avoidance of testing before low-risk surgery. The type of surgery is an important determinant of the risk of cardiac complications and mortality. In patients undergoing noncardiac surgery, functional status, generally defined in terms of metabolic equivalents (METs), is a reliable predictor of both perioperative and long-term risk [38].

The Lee index [39] is widely used for assessment of cardiac risk, because it is simple and has been extensively validated. However, more recent measures, such as that of Alrezk et al. [40], may be more accurate in older patients. The ACS-NSQIP Surgical Risk Calculator [41] has been specifically validated in geriatric patients, and is an accurate tool for preoperative assessment in this population, especially if combined with cardiac biomarkers [42].

The risk of postoperative venous thromboembolism is increased in patients over 70 years of age, and in geriatric patients with comorbidities such as cardiovascular disorders, malignancy or renal insufficiency. Therefore, risk stratification, correction of modifiable risks, and sustained perioperative thromboprophylaxis are essential in these populations. The timing and dosing of thromboprophylaxis in older patients should be the same as in younger patients [43].

#### Respiratory

**Table:** 

Statement	Quality of evidence	Strength of recommendation
We recommend that risk factors for respiratory complications be assessed and reduced where possible (e.g., abstention from smoking, optimization of therapies)	Moderate	A
We suggest the use of risk scores for postoperative pulmonary complications (ARISCAT)	Moderate	B

Postoperative pulmonary complications (PPCs) are common in geriatric patients, and contribute to the risks of perioperative and postoperative morbidity and mortality. The surgical site is the most important predictor of pulmonary complications; others include COPD, recent smoking, poor general health status, and functional dependency [44]. Age is a minor risk factor after adjustment for comorbidities, conferring an approximately twofold increase in risk [45]. Thus, older patients who are otherwise acceptable surgical candidates should not be denied surgery solely on the basis of concern about potential PPCs [46].

Routine preoperative spirometry is not recommended before high-risk surgery, because it is no more accurate in predicting risk than clinical evaluation. Patients who might benefit from preoperative spirometry include those with unexplained dyspnea or exercise intolerance, and those with COPD or asthma in whom the extent of airflow obstruction is unknown. A seven-variable risk assessment tool has been developed by the ARISCAT (Assess Respiratory Risk in Surgical Patients in Catalonia) Group [47], and prospectively validated [48].

Strategies for reducing the risk of PPCs in older surgical patients include risk factor minimization or avoidance (including preoperative smoking cessation), optimization of COPD or asthma treatment, deep breathing exercises, and epidural local anesthesia [46, 49]. In a general population of patients scheduled for elective upper abdominal surgery, a 30-min preoperative physiotherapy session provided as part of an existing multidisciplinary preadmission evaluation was shown to halve the incidence of PPCs, particularly hospital-acquired pneumonia [50].

#### Hematological

**Table:** 

Statement	Quality of evidence	Strength of recommendation
We recommend that a Patient Blood Management (PBM) strategy be implemented, including hemoglobin and iron optimization, predeposit autologous blood collection, and surgical and anesthetic strategies that reduce blood loss	Moderate	A
We recommend that hemoglobinemia be assessed in all geriatric patients, with particular attention to those aged > 80 years	Moderate	A
We recommend assessment of hemoglobinemia and iron metabolism in all geriatric patients who are candidates for surgery with expected blood loss > 500 ml, or who have fatigue, neoplastic disease, associated cardiovascular, respiratory or renal disease, resting tachycardia, or pallor	Moderate	A

Anemia is common in surgical patients, and is associated with increased perioperative mortality [51]. Preoperative anemia should, therefore, be considered a significant medical condition, rather than as simply an abnormal laboratory finding [52]. Preoperative anemia and iron deficiency are indications for a care pathway extending from the decision to operate until complete recovery, and anemia should be investigated before all surgical procedures with moderate‐to‐high anticipated blood loss (> 500 ml) [53]. Investigation should begin with an assessment of iron status: when ferritin or iron saturation levels indicate an absolute iron deficiency, referral to a gastroenterologist may be indicated to exclude gastrointestinal malignancy as a source of chronic blood loss. In the absence of an absolute iron deficiency, measurement of serum creatinine and glomerular filtration rate (GFR) may indicate chronic kidney disease (CKD) and the need for referral to a nephrologist. When ferritin or iron saturation values are inconclusive, further evaluation is necessary to exclude inflammation or chronic disease. A therapeutic trial of iron would confirm absolute iron deficiency, whereas a lack of response would indicate anemia of chronic disease, suggesting that treatment with an erythropoietin-stimulating agent should be initiated [54]. Iron-deficiency anemia should be treated with iron supplementation [55]. Oral iron replacement should be targeted to patients with iron deficiency with or without anemia whose surgery is scheduled 6–8 weeks after diagnosis [53].

Anemia and transfusion are associated with increased morbidity and mortality in surgical patients [56]. PBM (Patient Blood Management) is a multimodal, multidisciplinary, strategy aimed at minimizing the use of blood products and improving patients’ outcomes [57]. This strategy involves optimizing hemoglobin levels, minimizing perioperative blood loss, and optimizing the patient’s physiological reserve to withstand anemia [58]. PBM should be started before surgery, and continued throughout the perioperative period. Systematic preoperative PBM has consistently been shown to improve postoperative clinical outcomes [56, 59]. Maintenance of a preoperative hemoglobin level above 12.0 g/dl is recommended in older orthopedic patients, to reduce the need for perioperative blood transfusion [60]. Intraoperative PBM includes monitoring anemia and related physiological changes, conserving autologous blood, and using surgical and anesthetic strategies to contain and minimize blood loss. During the postoperative period, monitoring of anemia, organ perfusion, blood loss, and hemostasis is an important part of clinical management [58].

#### Renal

**Table:** 

Statement	Quality of evidence	Strength of recommendation
We recommend accurate estimation of renal function by calculating eGFR using the CKD-EPI equation	Moderate	A

The age-related decline of renal function varies markedly, due to nephrotoxic effects of comorbidities such as hypertension or diabetes, and drug treatment, particularly with non-steroidal anti-inflammatory drugs (NSAIDs) and angiotensin -converting enzyme (ACE) inhibitors. Renal impairment can affect anesthetic pharmacokinetics and pharmacodynamics, and hence, renal function should be assessed before any surgery in older patients [36]. The widely used Chronic Kidney Disease-Epidemiology Collaboration (CKD-EPI) equation for measuring estimated glomerular filtration rate (eGFR) is appropriate for use in this population.

#### Nutritional

**Table:** 

Statement	Quality of evidence	Strength of recommendation
We recommend evaluation of nutritional status and correction of any deficiency, especially before major surgery	Moderate	A
We recommend that albuminemia be assessed in all older surgical patients, especially those with hepatic comorbidity, multiple comorbidities, recent major pathology or suspected malnutrition, or candidates for major surgery	Moderate	A
In candidates for major surgery with organ failure, we recommend an estimation of hydration and volume status with an instrumental method (e.g., ultrasound estimation or bioimpedentiometry)	Moderate	A
We recommend preoperative and postoperative nutritional support (enteral or parenteral), according to the level of undernutrition and/or feeding possibilities for patients at severe nutritional risk	Moderate	A

Increasing age is often associated with an unhealthy nutritional status [61], which is a risk factor for postoperative complications [36, 62]. However, poor nutritional status is often insidious and, hence, often goes unrecognized. Assessment of nutritional status is, therefore, essential in older surgical patients [63, 64]. European Society for Clinical Nutrition and Metabolism (ESPEN) guidelines emphasize the importance of screening for malnutrition on admission or first contact, observation and documentation of food intake, regular assessment of weight and body mass index (BMI), and nutritional counseling [65].

Preoperative albumin levels predict postoperative outcomes [65‒67]. A preoperative serum albumin concentration of 3 g/dl may be considered to indicate severe nutritional risk, as may a weight loss of > 10‒15% within 6 months, or a BMI < 20 kg/m^2^. Patients at high nutritional risk before elective surgery should be managed with a multimodal approach including evidence-based interventions to optimize nutritional status, and surgery should be postponed if possible [68].

#### Medication

**Table:** 

Statement	Quality of evidence	Strength of recommendation
It is recommended that the pharmacological history must be extended to include all drugs used by the patient, including over-the-counter and herbal medicines	Low	A
If the patient is taking inappropriate medications (e.g., according to STOPP criteria), we recommend prudent withdrawal of these medications	Low	A

In one study, 55.3% of surgical patients aged ≥ 65 years received a potentially inappropriate medication in hospital [69], highlighting the need to recognize such inappropriate medications in the perioperative period. A comprehensive medication review, including over-the-counter medications, vitamins, and herbal supplements, is essential to identify medications that should be continued during the perioperative period, and potentially harmful, ineffective, or contraindicated medications [16]. This may be particularly important in patients experiencing, or at increased risk of, delirium, because any medication change could trigger delirium [70]. Treatment with anticholinergics and poly-medication are considered predisposing risk factors for POD; because anticholinergic compounds are present in a number of medications frequently prescribed to older patients for different purposes, the total anticholinergic burden should be considered in patients at risk of POD [71].

ACS/AGS Best Practices Guidelines [72] recommend that the immediate preoperative period is an ideal time to confirm that a plan for the perioperative management of nonessential chronic medications is in place. A number of validated tools are available to identify potentially inappropriate medications among older patients, including the updated Beers criteria [73] and the Screening Tool of Older Persons’ Prescriptions (STOPP) and Screening Tool to Alert to Right Treatment (START) criteria [74].

#### Delirium

**Table:** 

Statement	Quality of evidence	Strength of recommendation
To reduce the incidence of postoperative delirium, we recommend: • Identifying predisposing and precipitating risk factors early • Adapting surgical and anesthetic techniques • Avoiding medications that promote postoperative delirium Using opioid-free anesthesia or low-dose opioid anesthesia • Monitoring for delirium (CAM, 4AT)	Moderate	A

Delirium is an acute fluctuating alteration of mental state, reduced awareness, and disturbance of attention, that may be triggered by acute medical illness, surgery, trauma, or drugs [75, 76]; multiple factors may be present in an individual patient [77, 78]. Delirium is independently linked with poor postoperative outcomes, including medical complications, falls, prolonged hospitalization, permanent cognitive dysfunction, need for institutionalization, and death, and can cause significant patient and care giver distress. It is frequently missed in routine clinical care [79, 80]. European Society of Anaesthesiology (ESA) guidelines recommend that the preoperative evaluation should include identification of potential risk factors for postoperative delirium, to identify patients at high risk [70, 81]. If a specialist geriatrician is not available, preoperative screening for delirium risk should be performed by anesthetists, and the best strategic approach determined by the multidisciplinary team [82].

The Confusion Assessment Method (CAM) may be useful in screening for delirium [79], but sensitivity and specificity vary markedly, possibly due to the need for users to have training and knowledge of delirium. The Arousal, Attention, Abbreviated Mental Test 4, and Acute change Test (4AT) is brief and easy to use without specific training, and has wide applicability in various clinical settings [83]. It offers good sensitivity and patient completion rates [84], and can also be used to assess older patients presenting in emergency departments [83].

In patients over 60 years of age, avoiding episodes of deep anesthesia during surgery lasting more than 1 h can significantly reduce the risk of postoperative delirium. To avoid excessively deep anesthesia, guidelines from the UK recommend that depth of anesthesia should be monitored in all patients in this age group [77, 78].

Several classes of medication can increase the risk of delirium, and hence, medication review can decrease rates of delirium [77, 78]. Opioids can cause delirium, but remain vital for treating pain, which, in itself, can precipitate delirium. The perioperative analgesia plan must consider multimodal strategies and reducing the use of opioids: older patients have increased sensitivity to opioids, and need individual dose titration [85].

#### Pain

**Table:** 

Statement	Quality of evidence	Strength of recommendation
Every older patient should undergo a standardized pain history and physical examination For patients with cognitive disorders, we recommend the use of specific scales (PAINAD, NOPPAIN) for the evaluation of pain	Low	A

Older patients should undergo a standardized pain history and physical examination, to determine an appropriate analgesic plan [72]. Because cognitive impairment alters the experience of pain, the ability to communicate that experience, and the medical management of pain [86], specific behavioral scales have been developed to assess pain in cognitively impaired older adults [87]. These include the Non-communicative Patient's Pain Assessment Instrument (NOPPAIN) [88], and the Pain Assessment in Advanced Dementia (PAINAD) [89].

### Emotional status

**Table:** 

Statement	Quality of evidence	Strength of recommendation
We suggest screening for depression using validated scales (e.g., the Geriatric Depression Scale), and treatment where possible	Low	B
Where possible, we suggest preventive counseling (goal setting, advanced directives) in selected cases	Low	B

Approximately 15%–20% of adults aged ≥ 65 years are affected by depression, and the prevalence among preoperative geriatric patients is even higher [61]. Depressive symptoms are associated with poor functional recovery and increased likelihood of institutionalization after discharge [90]. Similarly, patients with preoperative anxiety and depressive symptoms have worse patient-reported outcomes than those without [91]. Patients with preoperative depressive symptoms are also more likely to develop POD [92]. The ACS-NSQIP/AGS guidelines strongly recommend screening patients for depression prior to surgery [13]; simple tools, such as the short (15-item) version of the Geriatric Depression Scale, can be used for this purpose [93].

### Social support

**Table:** 

Statement	Quality of evidence	Strength of recommendation
It is recommended that the availability of family and social support be investigated during the preoperative assessment to allow planning of substitutive support measures	Low	A

Social support predicts 30-day postoperative morbidity when included in a geriatric preoperative assessment [94]. Assessment of the patient’s social support network is, therefore, recommended in the ACS-NSQIP/AGS guidelines [13]. If there is concern about insufficient family or social support, preoperative referral to a social worker should be considered [13].

## Intraoperative management

### Positioning

**Table:** 

Statement	Quality of evidence	Strength of recommendation
When positioning an older patient on the operating table, we suggest that attention be paid to conditions of the skin (e.g., atrophy, injury) and the musculoskeletal system (e.g., bone deformities, joint stiffness, and presence of prostheses)	Low	B
It is recommended that positioning be adjusted according to the patient's problems, taking care to place adequate padding at bony prominences	Low	A

In older adults, the risk of peripheral nerve damage and pressure injuries resulting from malposition is increased by skin atrophy and decreased skin integrity [72]. Measures should, therefore, be taken to ensure proper positioning, taking into account musculoskeletal conditions such as kyphoscoliosis, and padding of bony prominences [36, 72]. Care should be taken when transferring the patient between their bed and the operating table, and when removing adherent items such as surgical dressings [36].

### Anesthesia

#### Type of anesthesia

**Table:** 

Statement	Quality of evidence	Strength of recommendation
It is recommended that the choice of anesthesia (technique/drugs/dosage) be individualized based on the characteristics of the patient and the type of intervention, to reduce the incidence of postoperative delirium and facilitate recovery	Moderate	A

There is insufficient evidence to recommend a single anesthetic plan for all older adults. The use of regional anesthesia as the primary modality may be beneficial in reducing perioperative mortality or major complications in patients undergoing surgery with intermediate or high cardiac risks [95]. In accordance with Enhanced Recovery After Surgery (ERAS) principles, combining neuraxial or regional techniques with general anesthesia results in less intra- and postoperative metabolic derangement and better control of postoperative pain.

#### Dosage

**Table:** 

Statement	Quality of evidence	Strength of recommendation
We recommend dose adjustment to avoid overdose, adverse hemodynamic effects, or inadequate depth of narcosis	Moderate	A
For induction and maintenance of general anesthesia with propofol, we recommend that the dosage be reduced by 20–50% in older patients	Moderate	A
For halogenated anesthetics, we recommend that the minimal alveolar concentration be calculated according to patient age	Moderate	A
Because the effect of anesthetics on the central nervous system is age-dependent, we recommend that halogenated and intravenous anesthetic dosages be modulated using an anesthesia depth monitoring system	Moderate	A

Age-related alterations in anesthetic pharmacokinetics and pharmacodynamics render older patients prone to unintentional overdosing [96], and appropriate dose adjustment is therefore essential. Particular care should be taken with hypnotic agents, because the dose required to induce anesthesia in older patients is lower, and the onset time longer, than in younger patients [97].

Propofol is suitable for older patients because of the rapid recovery time, and favorable adverse event profile. In patients older than 80 years, propofol is associated with less post-anesthetic cognitive impairment than other hypnotic agents [98]. However, the brain becomes more sensitive to the effects of propofol with age, and the elimination rate decreases linearly in patients older than 60 years; furthermore, geriatric patients are more sensitive to adverse effects of propofol, such as hypotension, compared with the general population [99, 100]. Therefore, the dose should be reduced in older patients, particularly when administered with other induction agents [98, 99]. Opioids have useful analgesic properties, but can cause delirium. Older patients have increased sensitivity to opioids, necessitating individual dose titration [85].

Depth of anesthesia monitors are recommended for patients at higher risk of adverse outcomes, irrespective of the anesthetic used [101]. With inhalational anesthetics, older patients require a lower minimal alveolar concentration (MAC) of anesthetic to achieve the same effect as in younger adults or children [102], and hence, it is recommended that the MAC for halogenated anesthetics should be calculated based on the patient’s age [103]. If depth of anesthesia monitoring is unavailable, iso-MAC charts should be used to calculate the dose according to age-adjusted MAC values [36, 103].

#### Depth of anesthesia monitoring

**Table:** 

Statement	Quality of evidence	Strength of recommendation
During general anesthesia, we recommend EEG-based monitoring to avoid excessive anesthesia depth, which is associated with increased risk of postoperative delirium	High	A
It is recommended that EEG-based monitoring is extended to procedures performed under sedation	High	A

Depth of anesthesia monitoring to avoid episodes of deep anesthesia can significantly reduce POD in patients aged over 60 years under general anesthesia for surgery lasting more than 1 h [104, 105]. Therefore, guidelines from the UK recommend that depth of anesthesia should be monitored in all patients aged over 60 years [77, 78].

It is not possible to titrate anesthetics on the basis of on-line electroencephalography (EEG) [106]. By contrast, brain monitoring using processed EEG, such as the bispectral index (BIS), facilitates anesthetic titration and reduces episodes of deep levels of anesthesia [104, 107]. Observational studies suggest a significant association between anesthesia depth, as measured by BIS, and long-term (≥ 1 year) mortality [108]. BIS monitoring may also prevent awareness phenomena related to light anesthesia levels, although these are rare events in older patients [109, 110].

Brain monitoring should also be extended to procedures performed under sedation. In a subgroup analysis of a randomized study, limiting sedation levels resulted in a reduction in POD in patients with low comorbid state [111]. However, the benefits of lighter sedation levels may be confounded by baseline comorbidities that increase the risk of delirium.

### Residual postoperative neuromuscular block

**Table:** 

Statement	Quality of evidence	Strength of recommendation
In patients undergoing general anesthesia with neuromuscular block, we recommend that neuromuscular function be monitored quantitatively, and its complete recovery (train-of-four ratio > 0.9) be facilitated at the end of the intervention	Moderate	A
We recommend that residual neuromuscular block always be antagonized	Moderate	A
We recommend the use of sugammadex when complete and fast recovery of rocuronium-induced neuromuscular block is required	Moderate	A
We recommend the use of sugammadex when anticholinesterases are ineffective for reversing rocuronium-induced neuromuscular block	Moderate	A

#### Neuromuscular blocking agents

Aging significantly affects the pharmacokinetics of neuromuscular blocking agents (NMBAs), particularly with drugs eliminated by hepatic or renal metabolism [112], and older patients are more sensitive to NMBAs than younger patients [113]. The intermediate-acting relaxants, vecuronium and rocuronium, which depend on end-organ elimination, may have a significantly prolonged duration of action in older patients, and appropriate dose adjustment is necessary [113]. Mivacurium action is also prolonged, due to age-related decreases in plasma acetylcholinesterase activity. Atracurium and cisatracurium, which are eliminated primarily by temperature-dependent, spontaneous Hoffman degradation, do not require dose adjustments in older patients, because the recovery time is almost identical to that in younger patients, although onset time may be delayed.

Monitoring the depth of neuromuscular blockade is essential in older patients to ensure appropriate dosing of NMBD during anesthesia and to avoid incomplete recovery from neuromuscular blockade after surgery. A train-of-four (TOF) > 0.9 has been advocated as a criterion for adequate reversal of neuromuscular blockade [113, 114].

#### Neuromuscular blockade reversal in older patients

Complications related to postoperative residual curarization (PORC) are more frequent in older patients than in younger patients [109]. Furthermore, pharyngeal function, which is often impaired in geriatric patients, may be worsened by PORC, possibly resulting in postoperative aspiration-induced pneumonia [115]. PORC is strongly linked to inadequate reversal of neuromuscular blockade, and hence, accurate dosing of NMBAs, vigilant monitoring of neuromuscular blockage, and appropriate administration of reversal agents, are essential.

The duration of action of the cholinesterase inhibitors neostigmine and pyridostigmine is prolonged in aged patients [116, 117], whereas this is not true for edrophonium [118]. Hence, neostigmine and pyridostigmine are preferable to edrophonium for reversal of neuromuscular blockade in older patients, because their prolonged duration of action can counteract age-related increases in the duration of action of NMBAs.

Sugammadex binds to aminosteroidal NMBAs (rocuronium and vecuronium), forming a complex that is primarily excreted by the kidneys. Decreases in kidney function reduce sugammadex clearance, and prolong its half-life and duration of action [119]. Sugammadex is significantly faster than neostigmine in reversing neuromuscular blockade, and is more likely to be associated with higher TOF values and a lower risk of PORC [120]. Reversal of NMB with sugammadex is rapid, although slightly slower than in younger adults [119], especially with low-dose sugammadex [121].

### Temperature control

**Table:** 

Statement	Quality of evidence	Strength of recommendation
We recommend body-temperature monitoring and active warming of the patient, preferably with a forced-air system, during the pre-, intra-, and postoperative periods	High	A
If forced-air heating is only partially efficacious (e.g., during prolonged open abdominal surgery), we suggest that warm intravenous fluids be administered	High	B

Most anesthetics can inhibit thermoregulation, resulting in perioperative hypothermia (< 36.0 °C). Older patients are particularly predisposed to hypothermia due to altered thermoregulation resulting from decreases in muscle mass, metabolic rate, and vascular reactivity [72]. Active warming procedures are, therefore, necessary to maintain normothermia. These may include covering the patient's head and body, increasing the ambient room temperature, warming intravenous and irrigating solutions, and applying external warming devices.

Core temperature should be monitored in operations lasting more than 30 min, and warming should be used in older patients [72]. A forced-air warming system set at 42 °C is the most effective means of rewarming older patients with postoperative hypothermia [122], but a prewarmed infusion could also reduce the incidence of perioperative hypothermia and improve outcomes in geriatric patients [123].

### Fluid and electrolyte balance

**Table:** 

Statement	Quality of evidence	Strength of recommendation
We recommend adequate monitoring to maintain a “near-zero” fluid balance	High	A

Appropriate use of intravenous fluids is important to prevent hypovolemia or dehydration in geriatric surgical patients. Multiple international guidelines allow unrestricted intake of clear fluids up to 2 h before elective surgery [124]. However, some recent guidelines advocate more restrictive fluid management [125]; the term “zero balance” has been introduced to describe a restrictive regimen aiming to avoid postoperative fluid retention [126]. Concerns remain about impaired organ perfusion with this restrictive approach. In one study, patients managed with a restrictive fluid approach had a significantly higher risk of acute kidney injury (AKI) than those receiving liberal fluid administration [127].

In ERAS guidelines, the preoperative goal is to prepare a hydrated, euvolaemic patient by avoiding routine mechanical bowel preparation and encouraging patients to drink clear liquids up to 2 h before induction of anesthesia. The intra-operative goal is to achieve a ‘zero’ fluid balance after uncomplicated surgery; goal-directed fluid therapy (GDT) is recommended for poorly prepared or high-risk patients or those undergoing more complex surgery. The postoperative goal is early transition from intravenous to oral fluid therapy [125]: GDT reduces postoperative mortality, compared with standard hemodynamic therapy, even in high-risk patients [128]. Recent Italian guidelines [129] recommend perioperative GDT to reduce morbidity, and possibly mortality in high-risk patients.

### Blood transfusion

**Table:** 

Statement	Quality of evidence	Strength of recommendation
We recommend that transfusion in geriatric patients follows a restrictive transfusion strategy (red blood cell transfusion threshold: Hb < 8 g/dl)	High	A
We recommend red blood cell transfusion when symptoms of intraoperative hypoxia and/or lactic acidosis and hemorrhage are present, regardless of the severity of anemia	High	A

The patient’s bleeding tendency must be assessed before surgery, irrespective of medical history or medication use and surgery scheduled according to optimization of erythropoiesis. Intraoperative PBM includes: meticulous hemostasis and surgical technique; cell salvage; appropriate use of drugs and hemostatic agents; point-of-care tests for bleeding; and restrictive transfusion thresholds [58].

The standard treatment for severe perioperative anemia is transfusion of allogenic red blood cells (RBC): more than 50% of such transfusions are given to old and frail patients [130]. Compensatory mechanisms for anemia are severely impaired in older patients, and this may result in greater vulnerability to anemia-related ischemic events and perioperative complications [54]. In the absence of specific clinical conditions reducing tolerance of anemia, restrictive rather than liberal, transfusion strategies are recommended [131]. In a study of older adults (age ≥ 70 years) undergoing hip fracture surgery, a restrictive transfusion strategy was found to be safe, and was associated with fewer cardiovascular complications but more transfusions, compared with a more liberal regimen [132]. The LIBERAL trial is currently evaluating the benefits of liberal transfusion strategies in older surgical patients [133].

RBC transfusions are always indicated if the hemoglobin concentration is < 6 g/dl, and may be indicated in patients with concentrations between 6 and 10 g/dl. RBC transfusions are not indicated in adult, hemodynamically stable, patients with hemoglobin > 7 g/dl, although a threshold of 8 g/dl may be applied to patients undergoing orthopedic or cardiac surgery, and patients with cardiovascular disease [58]. In a study in patients aged ≥ 65 years undergoing major noncardiac surgery, intraoperative blood transfusion was associated with decreased 30-day postoperative mortality, compared with patients who did not receive transfusions, among the subgroups with substantial operative blood loss or low preoperative hematocrit (< 24%). Conversely, transfusion was associated with increased mortality in patients with preoperative hematocrits between 30 and 35.9% or < 500 ml blood loss during surgery [134].

### Minimally invasive surgery

**Table:** 

Statement	Quality of evidence	Strength of recommendation
We suggest using minimally invasive techniques in older patients, to reduce the endocrine/metabolic response to stress and improve postoperative outcomes	Moderate	B
During laparoscopy, we recommend Avoiding exaggerated or prolonged Trendelenburg or anti-Trendelenburg positions Avoiding unjustified prolongation of surgical times Using the lowest possible intraperitoneal pressure (< 12 mmHg), to minimize the negative cardiovascular and respiratory effects caused by pneumoperitoneum Administering deep neuromuscular blockade, to allow the use of low working pressures	Moderate	A

Where feasible, laparoscopic surgery is becoming standard treatment for many common pathologies that disproportionately affect older patients. Benefits of laparoscopy include decreased postoperative pain, shorter hospitalizations, improved cosmesis, and a quicker return to normal activity. However, laparoscopy may be technically challenging, and imposes specific physiologic demands on older patients [135]. In a study in colorectal cancer patients aged ≥ 80 years, laparoscopic surgery was associated with less blood loss, fewer postoperative complications, and shorter hospitalizations, than open surgery [136]. A recent systematic review found that short-term outcomes after laparoscopic surgery for colorectal cancer were generally similar in older and younger patients, although the overall complication rate was slightly but significantly higher in older patients [137].

Recent years have seen the introduction of robot-assisted surgical techniques. These techniques appear to be safe in older patients, with no increased risk of death or morbidity compared with younger patients [138]. Nevertheless, their use should depend on the specific history and comorbidities of the individual patient [138].

## Postoperative care

### General strategies for optimizing postoperative recovery

**Table:** 

Statement	Quality of evidence	Strength of recommendation
In the postoperative period, we recommend: • Optimal postoperative pain control • Early mobilization and walking • Early resumption of feeding • Conservation of the sleep–wake rhythm • Reducing use of nasogastric tube and bladder catheters • Antithrombotic prophylaxis	Moderate	A
We recommend the use of Enhanced Recovery Pathways for older patients, according to the type of surgery performed	Moderate	A

ERAS protocols aimed at reducing postoperative morbidity cover the whole perioperative period [139]. This multidisciplinary approach decreases length of stay and healthcare costs by better postoperative pain and nausea control, integration of preoperative, intraoperative, and postoperative care, and education to enable patients and families to participate in care. In a systematic review of 24 studies, the ERAS items that most strongly predicted shorter hospitalization and lower morbidity were: absence of a nasogastric tube; early mobilization, oral nutrition, and removal of the urinary catheter; and use of nonopioid analgesia [140]. Importantly, reduction in surgical stress through ERAS appears to be particularly effective in reducing complications and supporting recovery in older and frail patients [141, 142].

### Postoperative delirium

**Table:** 

Statement	Quality of evidence	Strength of recommendation
It is recommended that prevention, recognition and treatment of postoperative delirium must be an objective of the multidisciplinary team	Moderate	A
We recommend that patients at risk for POD be monitored with validated diagnostic tools such as the CAM or 4AT, starting when they wake from anesthesia and continuing for 5 days thereafter	Moderate	A

POD assessment should be performed, while the patient is still in the recovery room, and repeated over 5 days. Validated screening tools, such as the 4AT, and the CAM [143] and its variant for use in intensive care (CAM-ICU), facilitate recognition of POD: diagnosis rates are almost 100% when screening tools are used, but low in their absence. We, therefore, recommend using POD screening tools in the postoperative period [144]. However, CAM requires specific training to ensure reliability of diagnosis, whereas the 4AT can be used in various care settings without specific training [147]. It also allows evaluation of patients who are unable to complete more detailed cognitive tests because of drowsiness or agitation. We, therefore, recommend that the 4AT is included in the preadmission assessment of older surgical patients, and if possible incorporated into nursing routines [144].

An Italian intersociety consensus has highlighted recommendations for the prevention, diagnosis, and treatment of delirium in older hospitalized patients [145]. All patients with delirium should receive an individualized treatment plan, including identification of underlying acute diseases and other clinical conditions that may lead to delirium. Medication reconciliation, early mobilization, promotion of physiologic sleep–wake rhythm, maintenance of adequate nutrition and hydration, and the provision of visual and auditory aids, should be implemented [78]. This requires a multidisciplinary, coordinated, approach, coordinated where possible by the geriatrician. It is also important to recognize the potential role of family and caregivers in supporting the patient. Flexibility of visiting hours, and the use of investigative tools to determine the patient’s needs and preferences, should be encouraged [146].

Cognitive changes after anesthesia also include postoperative neurocognitive disorders. Specific risk factors for such disorders should be evaluated in susceptible patients [148, 149].

### Postoperative nausea and vomiting

**Table:** 

Statement	Quality of evidence	Strength of recommendation
Because of the high risk (e.g., of aspiration pneumonia), we recommend the use of adequate prophylaxis against postoperative nausea and vomiting, and semi-Fowler's decubitus position	Low	A

Postoperative nausea and vomiting can cause fear and anxiety before surgery, and can result in poor patient satisfaction, prolonged time in the postanesthesia care unit, and unplanned hospital admission in surgical outpatients. Although younger age (< 50 years) is a primary risk factor for PONV, surgical factors and anesthetic techniques increase the risk, even in low-risk populations. In addition, approximately 15% of older patients are affected by dysphagia, and this may increase to 50% in those with neurologic disease; PONV in older patients with postoperative dysphagia can increase the risk of aspiration pneumonia [150].

Risk factors for PONV should be assessed in all older surgical patients. Patients at moderate or high risks should receive appropriate prophylactic interventions and risk mitigation strategies, according to guidelines and local practice [72].

### Postoperative pain

**Table:** 

Statement	Quality of evidence	Strength of recommendation
Personalized prevention and treatment of postoperative pain are mandatory. We recommend a multimodal approach or, when possible, locoregional or plane blocks (e.g., TAP block)	Low	A
We suggest encouraging use of the non-pharmacological components of the multimodal approach	Low	B

Inadequate analgesia for older surgical patients contributes to postoperative morbidity, including delirium, cardiorespiratory complications, and failure to mobilize. However, postoperative pain is poorly assessed and treated in older patients, particularly those with cognitive impairment [36]. Pain can be assessed using the numerical rating scale, visual analogic scale, or verbal rating scale in patients with mild-to-moderate cognitive impairment; we recommend the use of specific scales (PAINAD, NOPPAIN, and Doloplus-2) for those with severe cognitive disorders [145].

Analgesic plans for older adults should be multimodal to avoid adverse effects of opioid analgesics and anxiolytics [151]. Non-pharmacological methods (e.g., positioning, acupuncture, music therapy, massage) are important adjunctive analgesic modalities [69]. Benefits of this approach include better pain scores, reduced sedation frequency, and reduced usage of opioid medications, compared with systemic opioids alone [72]. Paracetamol is safe and should be considered first-line pain therapy, with only minor concerns about dosage (< 3 g/day in older patients) and the risk of renal insufficiency, compared with the general population. If paracetamol is ineffective, NSAIDs should be used at the lowest possible dose and for the shortest possible duration, with concomitant proton pump inhibitor therapy and monitoring for gastric and renal damage. Older patients are more sensitive to adverse effects of opioids and NSAIDs, and more prone to postoperative morbidity. The combination of opioid-free general anesthesia with neuraxial or regional local anesthesia, according to ERAS principles, is indicated in this situation [148]. Morphine is an effective analgesic for moderate or severe pain, but should be administered cautiously, particularly in patients with poor renal or respiratory function, cognitive impairment, or both [36]. Meperidine has consistently been associated with an increased risk of POD in older surgical patients, but the incidence of POD and cognitive decline with this agent appear to be similar to those seen with more frequently used postoperative opioids such as morphine, fentanyl, or hydromorphone [152].

Regional or neuraxial techniques, such as transversus abdominus plane (TAP) block, can decrease the need for intraoperative or postoperative systemic narcotics [153]. Patient-controlled analgesia can be considered in appropriate patients.

### Postoperative pulmonary complications

**Table:** 

Statement	Quality of evidence	Strength of recommendation
We recommend periodic evaluation of oxygen saturation and respiratory rate in the postoperative period	Moderate	A
We recommend that arterial blood gas analysis be used when conditions interfere with percutaneous oximetry (e.g., shivering, tremor, cold skin, hyperthermia, hypotension, advanced heart failure, high fever, atrial fibrillation, or other arrhythmias)	Moderate	A
We suggest that older patients should be treated with lung expansion techniques, such as deep breathing exercises, incentive spirometry or, when indicated, with non-invasive ventilation	Moderate	B

Postoperative pulmonary complications (PPCs) increase postoperative mortality, and health care costs [72, 154]. Older age may be an independent predictor of PPCs [155]. Compared with patients under 50 years of age, the incidence of PPCs is almost fivefold higher in those aged > 80 years [47]. Hence, periodic evaluation of oxygen saturation, arterial blood gases, and respiratory rate is recommended in older patients.

In addition to optimization of pulmonary status during the preoperative and intraoperative periods, several postoperative strategies can be used to prevent PPCs in older patients, including screening for signs and symptoms of dysphagia [155], incentive spirometry, chest physical therapy, and deep breathing exercises [44, 49]. Monitoring of vital signs in the post-anesthetic setting is essential to identify patients at potential risk of postoperative respiratory failure [156]. Incentive spirometry is widely used to prevent PPCs, although clinical effectiveness data are limited, and standardized protocols are lacking [157, 158].

### Postoperative cardiovascular complications

**Table:** 

Statement	Quality of evidence	Strength of recommendation
To prevent postoperative cardiac complications, we recommend monitoring (continuously in selected cases) and maintenance of cardiovascular measures (e.g., heart rate, arterial pressure) within baseline values	High	A
We suggest the use of graduated compression stockings or intermittent pneumatic compression, when indicated	High	B

Age has not consistently been found to be an independent predictor of perioperative cardiac risk, although perioperative mortality following acute myocardial infarction is higher in older than in young patients [159]. Strategies to reduce cardiac risk in older patients include the use of beta blockers and statins, perioperative blood pressure control, and preoperative ECG [159].

Thromboprophylaxis is usually based on low-molecular-weight heparins. Graduated compression stockings or intermittent pneumatic compression are a valuable alternative in selected situations, such as neurosurgery, and a useful complement in others, including some orthopedic procedures. Use of such measures in older surgical patients is endorsed in current ESA guidelines [43].

### Acute kidney injury

**Table:** 

Statement	Quality of evidence	Strength of recommendation
For prevention of renal damage, we recommend that adequate hydration be maintained, and hypovolemia and/or hypotension be avoided	High	A
We recommend caution in the use of potentially nephrotoxic drugs or drug combinations with a high risk of nephrotoxicity, and in the administration of contrast media for radiological procedures	High	A

In older patients, pre-existing CKD, male gender, diabetes mellitus, heart failure, and surgery are major risk factors for AKI, and both CKD and AKI are independently associated with poor surgical outcomes. The optimal approach to preventing AKI in older surgical patients is unknown, but perioperative close monitoring of fluid balance, avoidance of nephrotoxic drugs, appropriate adjustment of renally excreted drugs, careful use of contrast media, and prompt treatment of sepsis are appropriate in all older patients. Because relatively small changes in serum creatinine can predict or define AKI [160], estimation of GFR should be available prior to surgery to facilitate the early detection of AKI.

### Urinary tract infection

**Table:** 

Statement	Quality of evidence	Strength of recommendation
We recommend that urinary catheters be used only when essential, and be removed as soon as possible	High	A
We recommend to adopt strategies to prevent urinary tract infections before, during, and after insertion of a urinary catheter	High	A
We do not recommend complementary strategies (such as the use of alpha-blockers in men) to promote spontaneous urinary function after catheter removal	High	D

Older adults are at particular risk for urinary tract infection (UTI), particularly if immobilized [72]. Guidelines for the prevention and management of UTI recommend limited use of urinary catheters, aseptic insertion of catheters, and maintenance of a closed drainage system [161]. Clinical evidence suggests that early removal of urinary catheters, whenever possible, is related to a lower risk of urinary infection and faster hospital discharge [72, 162].

### Nutrition and liquid balance

**Table:** 

Statement	Quality of evidence	Strength of recommendation
It is recommended that older patients undergo daily assessment of caloric intake and water balance	Moderate	A
We recommended that swallowing should be evaluated, and the presence of oral lesions excluded in patients with signs and symptoms of dysphagia or a history of aspiration pneumonia	Moderate	A
We suggest that all older patients are seated during meals and for an hour after eating	Moderate	B
It is recommended that nutritional supplementation be given in patients with malnutrition or inadequate nutrition	Moderate	A
It is recommended that dental prostheses, if used, are readily available and easily accessible	Moderate	A

Several studies have highlighted the association between malnutrition and adverse outcomes in older patients [163], and systematic reviews have reported that early feeding in selected patients is not harmful [164‒166]. Thus, nutritional support should be continued from the preoperative period, or instituted early after surgery, to improve wound healing and recovery [36]. Enteral nutrition is associated with better outcomes (shorter hospitalization, reductions in incidence or severity of complications, and decreased healthcare costs), compared with parenteral nutrition [166].

Oral feeding ability and aspiration risk should be assessed daily in older patients. A dietary consultation should be initiated, and a formal swallowing assessment performed if indicated [72]. During oral feeding, the head of the bed should be elevated at all times, and the patient should be sitting upright while eating and for 1 h after each meal, to prevent aspiration [72]. In addition, fluid status should be evaluated daily, with recording of input/output or body weight, for at least the first 5 postoperative days [72].

Older persons undergoing hip fracture surgery are generally at risk of malnutrition due to the acute trauma and surgery-related anorexia and immobility. Voluntary oral intake in the postoperative phase is often inadequate in such patients, and hence, rapid deterioration of nutritional status and impaired recovery are common [166]. Thus, the ESPEN guidelines for geriatric patients recommend that older patients with hip fracture should be offered oral nutritional supplements postoperatively, to reduce the risk of complications [166]. Such nutritional support should be part of an individually tailored, multimodal, and multidisciplinary intervention to ensure adequate dietary intake, improve clinical outcomes, and maintain quality of life [166].

Early tube feeding is recommended for patients who are unable to take oral supplements, such as those undergoing major head and neck or neurosurgical procedures, or patients who are unable to take 50% of their caloric needs orally for more than 7 days [167].

### Pressure ulcers

**Table:** 

Statement	Quality of evidence	Strength of recommendation
Strategies to prevent and treat pressure injuries are recommended in patients at risk	Moderate	A

Hospitalized older patients, particularly frail patients with hip fractures [168], are at high risk of pressure ulcers. Health care teams should, therefore, assess the risk of pressure ulcers in all older postoperative patients, and should implement multimodal interventions to prevent and treat pressure ulcers, especially in at-risk patients [72].

### Surgical site infections

**Table:** 

Statement	Quality of evidence	Strength of recommendation
We recommend guideline-consistent antimicrobial prophylaxis in older patients, considering antibiotic pharmacodynamics and pharmacokinetics to avoid overdoses and drug-related adverse events	Moderate	A

Surgical site infections (SSIs) are associated with delayed wound healing, prolonged hospital stays, increased use of antibiotics, unnecessary pain, and (rarely) death. Antibiotic prophylaxis is a principal strategy for preventing SSIs, but reductions in SSIs can also be achieved by implementing multidisciplinary, hospital-wide, measures such as bowel preparation, skin preparation, disinfection and hygiene, maintenance of normothermia during surgery, and glycemic control [169].

In older patients, it is important to choose the antimicrobial agent according to the susceptibility profile of colonizing bacteria. Particular attention should also be paid to the dosing regimen, because the relationship between appropriately dosed preoperative antibiotics and reduced risk of SSIs is well established. However, older patients may have renal impairment necessitating dose adjustment [60, 170]. Guidelines recommend that preoperative antibiotics should be given within 60 min before surgical incision, and that the choice of agent should be based on the nature of the surgical procedure, risk factors, and the hospital’s unique pathogen profile [171].

### Hyperglycemia and glucose management

**Table:** 

Statement	Quality of evidence	Strength of recommendation
We recommend careful and prolonged assessment of blood glucose in older patients with or without diabetes Insulin is the treatment of choice for the management of hyperglycemias	Moderate	A
We suggest that blood glucose values up to 180 mg/dl are acceptable in critical postoperative patients	Moderate	B

In older patients, postoperative hyperglycemia is associated with poor wound healing, SSI, acute complications (fluid and electrolyte disorders, acute renal failure), longer hospitalization, and death [172]. Glucose management is, therefore, an important aspect of postoperative care, and insulin is the drug of choice to treat postoperative hyperglycemia [173‒175].

### Hospital discharge and continuity of care

**Table:** 

Statement	Quality of evidence	Strength of recommendation
During the surgical planning, we recommend evaluating the probability of the patient returning home at the end of the hospital stay	Moderate	A
For patients with functional deterioration and/or weak social networks, we recommend that the discharge plan, including outpatient visits and medications, should be organized in advance in collaboration with the geriatrician; ideally, a Geriatric Evaluation Unit should be available	Moderate	A
Before hospital discharge, we recommend re-evaluating and adjusting drug therapy according to home therapy, the surgery performed, comorbidities, and any new pathological changes	Moderate	A
We recommend that the patient and caregiver be provided with resources to deal with problems that may arise at home, and with patient management contacts and references (telephone/e-mail)	Moderate	A

The question of where the patient can receive the best possible support after discharge should be considered throughout the perioperative period. The lack of an appropriate discharge and transition plan makes early readmission more likely, and may impair functional status and quality of life [176]. It is essential to establish an organizational framework that incorporates: appropriate assessment of the patient’s clinical, social and care status; recognition of patients’, relatives’ and caregivers’ expectations; formal planning and coordination of discharge; good knowledge of transitional management programs; and communication between different care settings [177].

Changes to medication frequently occur during hospitalization of older adults, and prompt review within primary care is essential following discharge [178, 179].

CGA of frail geriatric patients can reduce the risk of readmission when performed immediately before hospital discharge or on arrival in community settings. This should include targeting criteria to identify vulnerable patients, a multidimensional assessment program, comprehensive discharge planning, and home follow-up.

Some frail patients may develop a transient period of health vulnerability following hospitalization, known as the post-hospital syndrome (PHS) [180]. PHS is characterized by the risk of early re-hospitalization due to physiologic stressors resulting from the initial admission, including disruption in sleep–wake cycles, inadequate pain control, deconditioning, and changes in nutritional status. Patients hospitalized within 90 days of elective surgery are at increased risk of PHS [181].

Geriatric patients, especially if frail, often need prolonged hospitalization, or care in intermediate care facilities, before returning home. For some patients, worsening health and functional status make it impossible to return home. In a study of surgical patients aged ≥ 65 years, 30% were discharged to an institutional care facility; the strongest predictors of institutionalization were a TUG time ≥ 15 s and functional dependence [182]. Discharge to residential care, and inability to maintain independence after surgery, may be unacceptable to many older patients [183]. Anticipating which adults will require discharge to care facilities is important for preoperative counseling and care planning for both patients and caregivers. Before surgery, patients and surgeons should discuss clearly what they hope to achieve with the intervention, and what secondary strategy should be adopted if these objectives are not achieved or complications occur. Furthermore, all members of the multidisciplinary team should take into consideration advance directives such as “do not resuscitate” orders. The patient’s autonomy must always be respected, and it should not be assumed that the patient will accept all postoperative treatments should complications occur [184].

## Conclusions

These recommendations should facilitate the multidisciplinary management of older surgical patients, integrating the expertise of surgeons, anesthetists, geriatricians, and other specialists and health care professionals. These roles might vary according to the phase and setting of care and the patient’s condition.

A number of general statements can be made about the perioperative care of geriatric surgical patients. First, prehabilitation and ERAS protocols are recommended in all older candidates for elective surgery. Second, continuity of care is the hallmark of optimal care, and this requires early planning of the expected needs, final location of care and transition strategies for problematic cases. Finally, for medium- to high-risk patients, implementation of CGA and associated care should be considered in terms of the relative costs and benefits, rather than cost alone.
