# Correction to: Perioperative Management of Elderly patients (PriME): recommendations from an Italian intersociety consensus

**DOI:** 10.1007/s40520-020-01701-1

**Published:** 2020-09-10

**Authors:** Paola Aceto, Raffaele Antonelli Incalzi, Gabriella Bettelli, Michele Carron, Fernando Chiumiento, Antonio Corcione, Antonio Crucitti, Stefania Maggi, Marco Montorsi, Maria Caterina Pace, Flavia Petrini, Concezione Tommasino, Marco Trabucchi, Stefano Volpato, Società Italiana di Anestesia Analgesia Rianimazione e Terapia Intensiva (SIAARTI), Società Italiana di Gerontologia e Geriatria (SIGG), Società Italiana di Chirurgia (SIC), Società Italiana di Chirurgia Geriatrica (SICG) and Associazione Italiana di Psicogeriatria (AIP)

**Affiliations:** 1 Fondazione Policlinico Universitario A. Gemelli IRCCS, Rome, Italy; 2 Università Cattolica del Sacro Cuore, Rome, Italy; 3 Policlinico Universitario Campus Biomedico, Rome, Italy; 4 Past Director Geriatric Surgery Area and Anaesthesia Dpt., INRCA, Italian National Research Centre on Aging, Ancona, Italy; 5 Università degli Studi di Padova, Padua, Italy; 6 ASL Salerno, Salerno, Italy; 7 Monaldi-Ospedale Dei Colli, Naples, Italy; 8 CNR, Institute of Neuroscience, Aging Branch, Padua, Italy; 9 Humanitas University and Research Hospital IRCCS, Milan, Italy; 10 Università degli Studi “Luigi Vanvitelli”, Naples, Italy; 11 Università degli Studi G.d’Annunzio, Chieti, Italy; 12 Università degli Studi di Milano, Milan, Italy; 13 Università degli Studi di Tor Vergata, Rome, Italy; 14 Università degli Studi di Ferrara, Ferrara, Italy

## Correction to: Aging Clinical and Experimental Research 10.1007/s40520-020-01624-x

The article Perioperative Management of Elderly patients (PriME): recommendations from an Italian intersociety consensus, written by Paola Aceto · Rafaele Antonelli Incalzi · Gabriella Bettelli · Michele Carron · Fernando Chiumiento · Antonio Corcione · Antonio Crucitti · Stefania Maggi · Marco Montorsi · Maria Caterina Pace · Flavia Petrini · Concezione Tommasino · Marco Trabucchi · Stefano Volpato, was originally published electronically on the publisher’s internet portal (currently SpringerLink) on 10 July 2020 without open access. With the author(s)’ decision to opt for open choice, the copyright of the article changed on 10 September 2020 to © The Author(s) 2020 and the article is forthwith distributed under the terms of the Creative Commons Attribution 4.0 International License (http://creativecommons.org/licenses/by/4.0/), which permits use, duplication, adaptation, distribution and reproduction in any medium or format, as long as you give appropriate credit to the original author(s) and the source, provide a link to the Creative Commons license and indicate whether changes were made. The original article has been corrected.

